# Supplementary figures and images for: Sensing ecosystem dynamics via audio source separation: A case study of marine soundscapes off northeastern Taiwan
Source: PLoS Comput Biol. 2021 Feb 18;17(2):e1008698. doi: 10.1371/journal.pcbi.1008698 (PMC7891715; doi:10.1371/journal.pcbi.1008698)

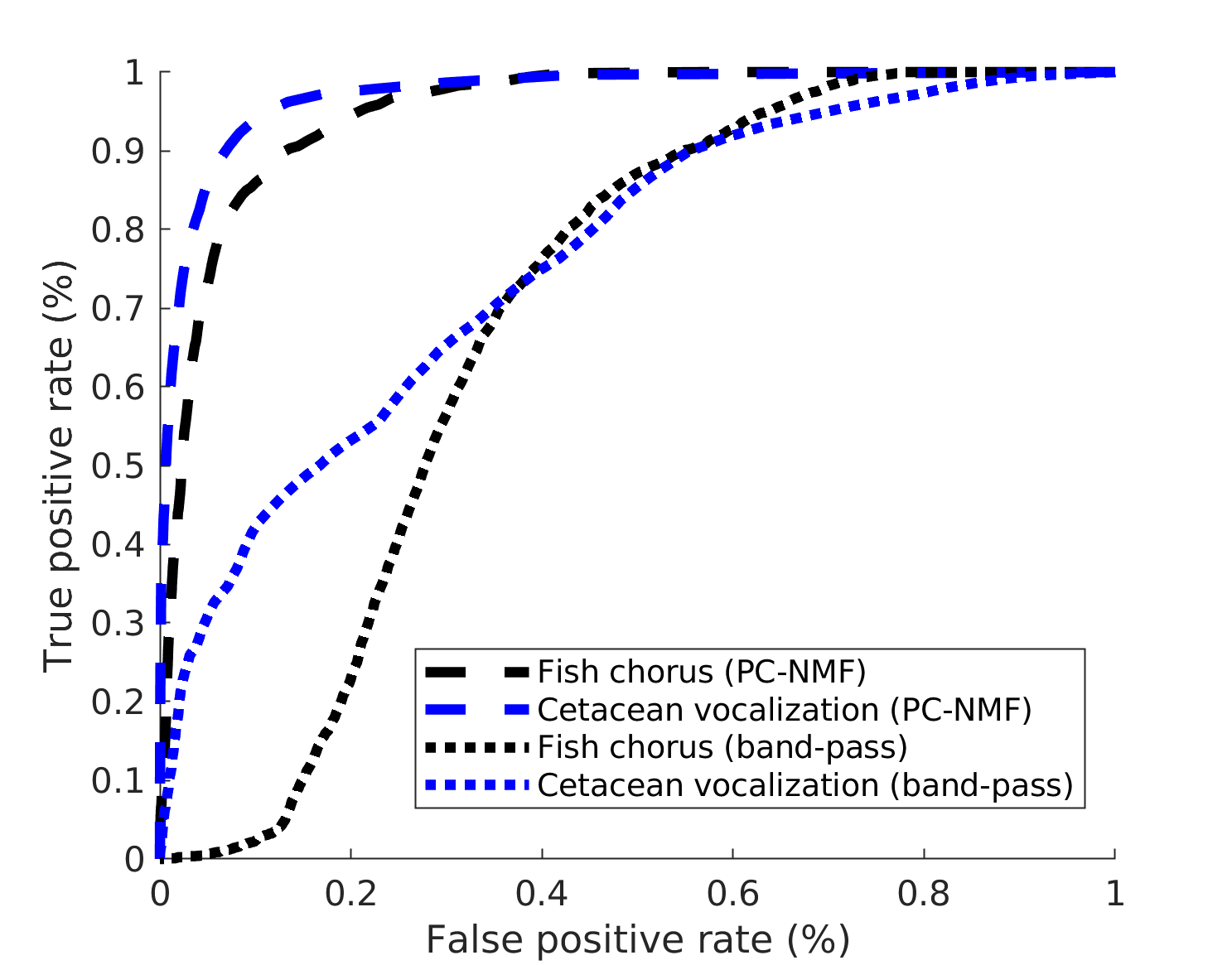

Supplement: S1 Fig — The performance of PC-NMF was evaluated by comparing the reconstructed LTSA of fish choruses and cetacean vocalizations to the manual identification results. In addition, we also ran source-specific band-pass filters (according to the spectral peaks identified in Fig 2) on the prewhitened LTSA. The PC-NMF can correctly detect more fish choruses and cetacean vocalizations than the conventional band-pass filters. (TIF) [file pcbi.1008698.s001.tif]

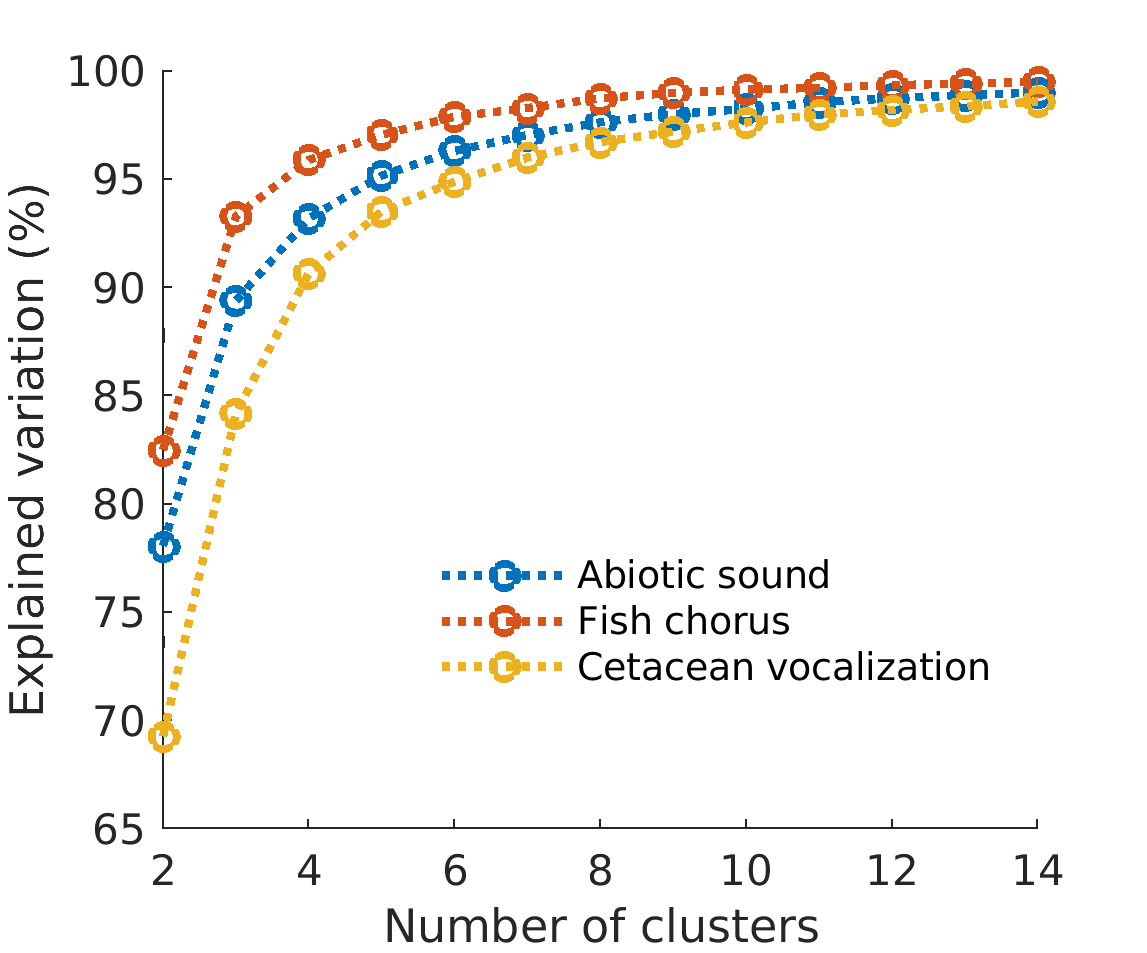

Supplement: S2 Fig — To compare the acoustic diversity among abiotic sounds, fish choruses, and cetacean vocalizations, we iteratively changed the number of clusters and measured the percentage of variation that can be represented by cluster centroids. The curves show that cetacean vocalizations require the highest number of clusters to explain the same level of variation as fish choruses and abiotic sounds. Fish choruses require the least number of clusters to explain the same variation as abiotic sounds and cetacean vocalizations. (TIF) [file pcbi.1008698.s002.tif]
