# Supplementary material for: Sensing ecosystem dynamics via audio source separation: A case study of marine soundscapes off northeastern Taiwan
Source: PLoS Comput Biol. 2021 Feb 18;17(2):e1008698. doi: 10.1371/journal.pcbi.1008698 (PMC7891715; doi:10.1371/journal.pcbi.1008698)
Supplement: Attachment — Submitted filename: review.pdf [file pcbi.1008698.s003.pdf]

The authors used source separation, a method commonly used in music science but innovative in ecoacoustics, to study the dynamics of the marine soundscape off Taiwan. This method is based on the principle that each sound source making up the composite soundscape has a specific periodicity pattern.

The authors analysed 2.5 years of audio data recorded by a single hydrophone. They were able to separate abiotic sounds, fish choruses, and cetacean sounds. They identified several cycles in the occurrence of fish choruses and cetacean sounds, as well as interactions between the occurrence of fish choruses and cetacean sounds. The authors further classified abiotic sounds, fish choruses, and cetacean sounds in several categories, which allowed them to describe changes in the composition of fish choruses and cetacean sounds throughout the seasonal cycles.

Introduction

The introduction describes the importance and challenges of studying ecosystemic trends and biodiversity changes in the ocean. The authors highlight audio recording as a powerful tool for the study marine ecosystems. They present commonly used methods and their downsides for the study of acoustic data.

The presentation of source separation as the method selected by the authors is succinct; I would have appreciated a more detailed description of source separation and its advantages over the other methods here.

Results

The Results section is complete and follows the authors' claims from the introduction. The authors first present the separation of abiotic sources, fish choruses, and cetacean sounds. Then, they describe the temporal dynamics of each source and investigate interactions between sources. They present the categorisation of each source into cluster and analyse the composition of each source during the diurnal and seasonal cycle.

The authors present many analyses in this section. The Results section is quite dense and may be confusing for the readers. To improve clarity, I would suggest moving the Material and Methods section before the Results, or using subheadings to guide the readers through the Results.

The authors mention that data were heavily corrupted in the high frequencies between March and July 2013 and that these data were discarded in subsequent analyses. However, there is no mention as to how these missing data are taken into account, for instance in the time-lagged correlation analyses. Were these data included in the precision rates and false positive rates presented line 141-142?

Line 142: “with a 5% false positive rate”. Is this an overall false positive rate, or do both fish choruses and cetacean vocalisations have a 5% false positive rate?

Line 143, Figure 1: This figure is a really nice visualisation of audio source separation. Could you add a colourbar to this figure to mention what is represented by the colours, what are the boundaries of the colouring, and what scale is used?

Line 149, Figure 2: What does “Relative amplitude” mean, and why does it vary between 0 and 50?

Line 183: Could you define what a “primary week” is, or reword this sentence? In addition, the decreased occurrence of cetacean sounds around week 47 (Figure 5) seems to undermine the claim that the “primary weeks” of cetaceans are week 35-17.

Line 194: “These clusters were associated with different types of vessel or vessel behaviors”. This sentence is a bit vague. Does it refer to different vessel sizes, propulsion systems, speeds?

Line 199, Figure 6: It is difficult to visualise the variations of each cluster in the stacked histograms. It may be more visible to plot each cluster separately to see the compositional shifts.

Discussion

The Discussion is well written. The authors summarise their results and emphasise the ability of source separation to distinguish simultaneous, interfering sources. They present the limitations of their method in terms of information retrieval. They discuss the future prospects of their method to study the effect of anthropogenic sounds on marine ecosystems, to assess changes in marine biodiversity. They mention leads to improve their model.

The authors managed to identify three clusters corresponding to different shipping noise. I would have liked to see these data used to discuss the impact of anthropogenic sounds on marine ecosystems.

I would have liked to see more effort into the identification of species corresponding to each cluster. Although it may be impossible within fish choruses, as the authors mention, it mey be easier for marine mammal species. Spectral features and temporal patterns of occurrence, in association with sighting data from the literature, may provide useful information for species identification.

Line 246-260: The first sentence of this paragraph does not match with its content. The authors state that the main advantage of the PC-NMF method is the unsupervised learning process, but they go on to explain how the intervention of experienced users in the learning process (therefore semi-supervised learning) could improve the model performance.

Line 273-274: Acoustic diversity only represents sound-producing individuals. Therefore, it may have limited use as a proxy of biodiversity.

Material and Methods

The Material and Methods section is pretty complete. It describes the recording material and study site in detail. The algorithm underlying the periodicity-coded non-negative matric factorisation has been published previously (Lin et al. 2017), and all the Matlab code used in this study is available from Code Ocean. However, some details in the analyses are missing.

Line 409, Figure 8: I would suggest changing the numbering of the spectral features of each source, or splitting spectral features by source to better see which temporal activations/periodicities correspond to which source.

Line 416: Why 90 spectral features? Was it an arbitrary choice or was it justified?

Line 428: Similarly, why 15 minutes and 0.5? Did you optimise the parameters?

Line 434-435: How did the first author determine whether a spectral feature was mislabelled?

Did you use absolute LTSA levels to compare the sound levels of the different sources?

Which criteria did you use in the time-lagged correlation analysis to define a recurring pattern?
