# Supplementary material for: Sensing ecosystem dynamics via audio source separation: A case study of marine soundscapes off northeastern Taiwan
Source: PLoS Comput Biol. 2021 Feb 18;17(2):e1008698. doi: 10.1371/journal.pcbi.1008698 (PMC7891715; doi:10.1371/journal.pcbi.1008698)
Supplement: Attachment — Submitted filename: ResponseLetter.docx [file pcbi.1008698.s004.docx]

**RESPONSE TO GUEST EDITOR**

**COMMENT:** *The reviewers all found the paper to be an interesting application of the authors' previously-developed PC-NMF method to a larger dataset of marine soundscape data. However, in order for this paper to be a substantial contribution to the literature, there needs to be either (a) a significant computational innovation (this paper does not aim to provide that, since it relies on the authors' previously-published technical method); or (b) a clear analysis of the results as a contribution to the biological understanding of the ecosystem considered. As reviewer 3 highlights, the submitted paper does not give much information of the existing state of knowledge about the ecosystem or species interactions in the northeastern Taiwan sea.* ***DONE.***

**REPLY:** We appreciate the constructive suggestions from the guest editor and the four reviewers. We believe that we have made the appropriate revisions according to the review comments.

**COMMENT:** *The paper needs a statement of the current (before the study) knowledge of the northeastern Taiwan soundscape, and further discussion of the biological findings of this study.* ***DONE.***

**REPLY:** The revised manuscript has added a section to review the current knowledge on the marine soundscapes off northeastern Taiwan (section of **Marine Soundscapes off Northeastern Taiwan**). We discussed the advantage of using PC-NMF over the other methods, including the capability of unsupervised feature learning (**Fig 8** in the revised manuscript) and the flexibility to develop a model in a semi-supervised approach (section of **Applying Audio Source Separation in Assessing Soundscapes**). We also added more paragraphs to demonstrate how applied techniques can improve acoustic diversity assessment and reveal the difference in spectral characteristics and phenological patterns between groups of marine soniferous animals (**Fig 7** in the revised manuscript). Furthermore, we discussed how the result of soundscape information retrieval could improve our understanding of the community of marine soniferous animals in a continental shelf environment and their interactions with other species and anthropogenic activities (section of **Retrieving Ecological Information from Soundscapes**).

**COMMENT:** *How exactly would someone access the data? The main website is given. Are there cataglogue numbers or specific IDs/URLs that could be used to access the data?* ***DONE.***

**REPLY:** All recording clips are archived in the Geophysical Database Management System (<http://gdms.cwb.gov.tw>). The data are only available for research use and require permission granted by the Central Weather Bureau of Taiwan. Despite that, we will publish a dataset of the long-term spectral average (LTSA) used in this project in depositar after this manuscript has been accepted for publication (<https://data.depositar.io/en/>). The previous statement has been included in the section of **Data Accessibility**.

**COMMENT:** *"two layers of non-negative matrix factorisation" is not an appropriate description, since the NMF algorithm appears to be applied only once.* ***DONE.***

**REPLY:** The PC-NMF is a tool of blind source separation based on two layers of NMF. The first layer is a feature learning layer. The second layer relies on the temporal activations learned by the first layer and determines the source indicators of spectral features (also learned in the first layer). The third reviewer may confuse the application of PC-NMF in different stages of data processing. Therefore, we have revised the section of **Audio Source Separation**. The revised content provided more details on the PC-NMF, and its usage in the model training phase, prediction phase, and reconstruction phase. In short, the model training phase used two NMF layers, but the prediction and reconstruction phases only used the first NMF layer.

**RESPONSE TO REVIEWER 1**

**COMMENT:** *This paper describe a promising approach to long term unlabeled acoustic recording analysis, with a successful application and interesting results.* ***DONE.***

**REPLY:** We appreciate the constructive suggestions from the reviewer. We believe that we have made the appropriate revisions according to the review comments.

**COMMENT:** *There is I believe a mistake line 239 with "contains" instead of "containing" ?* ***DONE.***

**REPLY:** We rewrote this sentence as “This study demonstrated that the assumption of source-specific periodicity is effective for the BSS of marine soundscapes because the PC-NMF can differentiate the different periodic patterns between biotic and abiotic sounds.” (**Line 317**)

**COMMENT:** *Figure 2 could be displayed as the curve of the median +- the std to avoid singular peaks? was this figure computed over the whole recording period?* ***NO CHANGE.***

**REPLY:** Yes, this figure (**Fig 3** in the revised manuscript) was computed over the entire dataset of MACHO recordings. The peak frequencies of 3.5, 43.4, and 50 kHz are likely the narrowband sounder signals generated by vessels. They are also the key acoustic features for the fifth cluster of abiotic sounds. If we changed the curve to display the median and the std, these features would not be effectively displayed.

**COMMENT:** *Insights could be added on why/how the algorithm works for abiotic sounds despite the fact that there are not periodic (as seen in l.160 and fig 3).* ***DONE.***

**REPLY:** We have discussed this point in **Line 317**: “This study demonstrated that the assumption of source-specific periodicity is effective for the BSS of marine soundscapes because the PC-NMF can differentiate the different periodic patterns between biotic and abiotic sounds.”

**COMMENT:** *Details on the clustering procedure, and for the rest of the method, could help for an easier read. The method is explained, but could use more details to enhance reproducibility.* ***DONE.***

**REPLY:** We have added more details on the methodology used, especially the Sections of **Audio Source Separation**, **Analysis of Acoustic Phenology**, and **Evaluation of Acoustic Diversity**. We believe that we have made the appropriate revisions according to the review comments.

**COMMENT:** *If I understood correctly, the sample size for the correct identification and false positive rates is 12 days (l.433)? times 24 hours times 2 LTSA per hour = 576? this could be clarified with the presented results.* ***DONE.***

**REPLY:** We subsampled 12 days of recordings to build the source separation model and conduct performance evaluation. Each hour has 120 recording clips because the duration of each audio clip is 30 sec. Excluding a small portion of missing files, the total amount of samples used in the model building and performance evaluation is 34,541 clips. (**Line 551**)

**COMMENT:** *Insights on the computation power / time needed depending on the size of the data used for learning could benefit the paper.* ***DONE.***

**REPLY:** The revised manuscript has added a section to discuss the computation power and time needed in model building. (**Lines 467-478**)

**RESPONSE TO REVIEWER 2**

**COMMENT:** *The introduction describes the importance and challenges of studying ecosystemic trends and biodiversity changes in the ocean. The authors highlight audio recording as a powerful tool for the study marine ecosystems. They present commonly used methods and their downsides for the study of acoustic data. The presentation of source separation as the method selected by the authors is succinct; I would have appreciated a more detailed description of source separation and its advantages over the other methods here.* ***DONE.***

**REPLY:** The revised manuscript introduced the difference between blind source separation (BSS) and clustering. We also discussed why the other methods (conventional bioacoustics approaches, supervised learning approaches, clustering, and previous works of BSS) might not perform well in the analysis of soundscapes (**Lines 99-128**). We also discussed the advantages of PC-NMF over the state-of-the-art deep learning methods (**Line 314-317**).

**COMMENT:** *The Results section is complete and follows the authors’ claims from the introduction. The authors first present the separation of abiotic sources, fish choruses, and cetacean sounds. Then, they describe the temporal dynamics of each source and investigate interactions between sources. They present the categorisation of each source into cluster and analyse the composition of each source during the diurnal and seasonal cycle. The authors present many analyses in this section. The Results section is quite dense and may be confusing for the readers. To improve clarity, I would suggest moving the Material and Methods section before the Results, or using subheadings to guide the readers through the Results.* ***DONE.***

**REPLY:** We appreciate the review’s suggestion. The revised manuscript added three subheadings in the Results.

**COMMENT:** *The authors mention that data were heavily corrupted in the high frequencies between March and July 2013 and that these data were discarded in subsequent analyses. However, there is no mention as to how these missing data are taken into account, for instance in the time-lagged correlation analyses. Were these data included in the precision rates and false positive rates presented line 141-142?* ***DONE.***

**REPLY:** The audio data from March 24 to July 1, 2013, recorded high-intensity broadband noise in frequencies ≥ 30 kHz, leading to incorrect detection of cetacean vocalizations. Because the high-frequency range was too heavily corrupted to identify echolocation clicks manually, the intensities of cetacean vocalizations were manually set to 0 to prevent bias in the phenological pattern analysis. (**Lines 223-228**). The performance evaluation was only based on the 12-days audio data selected from the first year. (**Lines 551-562**). The corrupted audio data were not included in the performance evaluation and the other parts of acoustical analyses.

**COMMENT:** *Line 142: “with a 5% false positive rate”. Is this an overall false positive rate, or do both fish choruses and cetacean vocalisations have a 5% false positive rate?* ***DONE.***

**REPLY:** Yes, the false positive rates were fixed at 5% for both fish choruses and cetacean vocalizations. The information was added in **Lines 194-200**.

**COMMENT:** *Line 143, Figure 1: This figure is a really nice visualisation of audio source separation. Could you add a colourbar to this figure to mention what is represented by the colours, what are the boundaries of the colouring, and what scale is used?* ***DONE.***

**REPLY:** We appreciate the review’s suggestion. The revised figure (**Fig. 2** in the revised manuscript) has a color bar for each panel.

**COMMENT:** *Line 149, Figure 2: What does “Relative amplitude” mean, and why does it vary between 0 and 50?* ***DONE.***

**REPLY:** The relative amplitudes represent the signal strength displayed on the prewhitened LTSA after performing source separation. This information has been included in the figure legend (**Fig 3** in the revised manuscript).

**COMMENT:** *Line 183: Could you define what a “primary week” is, or reword this sentence? In addition, the decreased occurrence of cetacean sounds around week 47 (Figure 5) seems to undermine the claim that the “primary weeks” of cetaceans are week 35-17.* ***DONE.***

**REPLY:** We rewrote this sentence as “In addition, the differences in the seasonal changes in intensities between fish choruses (highest at week 28) and cetacean vocalizations (highest at week 6) reflected the 186-day peak in the time-lagged correlations.” (**Line 249**)

**COMMENT:** *Line 194: “These clusters were associated with different types of vessel or vessel behaviors”. This sentence is a bit vague. Does it refer to different vessel sizes, propulsion systems, speeds?* ***DONE.***

**REPLY:** Following the reviewer’s suggestion, we rewrote this paragraph as “Abiotic sounds were separated into five clusters, which explained 95.17% of the variation (Fig 7). Among them, the first cluster was characterized by low-frequency sounds (< 2 kHz) and was recorded more frequently after July 2013. The second to fourth clusters were associated with the underwater radiated noise of moving vessels, and many events recorded 43.4 or 50 kHz narrowband sounder signals. The last cluster was characterized by narrowband sounder signals (peak frequency at 3.5 kHz or 43.4 kHz) and was mainly recorded from 5 AM to 10 AM, and from May to October.” (**Lines 263-270**)

**COMMENT:** *Line 199, Figure 6: It is difficult to visualise the variations of each cluster in the stacked histograms. It may be more visible to plot each cluster separately to see the compositional shifts.* ***DONE.***

**REPLY:** Following the review’s suggestion, each cluster was plotted separately (**Fig 7** in the revised manuscript). In addition, we also included an example of reconstructed LTSA for each cluster.

**COMMENT:** *The Discussion is well written. The authors summarise their results and emphasise the ability of source separation to distinguish simultaneous, interfering sources. They present the limitations of their method in terms of information retrieval. They discuss the future prospects of their method to study the effect of anthropogenic sounds on marine ecosystems, to assess changes in marine biodiversity. They mention leads to improve their model. The authors managed to identify three clusters corresponding to different shipping noise. I would have liked to see these data used to discuss the impact of anthropogenic sounds on marine ecosystems.* ***DONE.***

**REPLY:** Following reviewer’s suggestion, we added a section to discuss the potential impacts of sound masking due to the prevalent anthropogenic sounds recorded by the MACHO (**Lines 422-435**). In addition, we also discussed the future possibilities of using the detections of narrowband sounder signals to investigate the strategy of local fisheries (**Lines 446-453**).

**COMMENT:** *I would have liked to see more effort into the identification of species corresponding to each cluster. Although it may be impossible within fish choruses, as the authors mention, it may be easier for marine mammal species. Spectral features and temporal patterns of occurrence, in association with sighting data from the literature, may provide useful information for species identification.* ***DONE.***

**REPLY:** Identifying the species responsible for the cetacean vocalizations recorded by the MACHO is very challenging, because of the limited published articles on the acoustic behavior of cetaceans in this region. Despite that, we still provide a list of species commonly occurred in this region (**Lines 383-393**).

**COMMENT:** *Line 246-260: The first sentence of this paragraph does not match with its content. The authors state that the main advantage of the PC-NMF method is the unsupervised learning process, but they go on to explain how the intervention of experienced users in the learning process (therefore semi-supervised learning) could improve the model performance.* ***DONE.***

**REPLY:** We rewrote the section of **Applying Audio Source Separation in Assessing Soundscapes** to emphasize two primary advantages of using the PC-NMF. The first advantage is the unsupervised feature learning and the automatic recognition of source indicators according to the assumption of source-specific periodicity. This part was discussed in **Lines 327-348**. The second advantage is the visualization of model parameters, which allow experienced users to change the source indicators of spectral features and subsequently improve the unsupervised learning result. This part was discussed in **Lines 355-366**.

**COMMENT:** *Line 273-274: Acoustic diversity only represents sound-producing individuals. Therefore, it may have limited use as a proxy of biodiversity.* ***DONE.***

**REPLY:** We have changed the text as “Even without knowing the species identity, the observed acoustic phenology can help us evaluate the changes in the community structure and behavior over time.” (Line 394)

**COMMENT:** *The Material and Methods section is pretty complete. It describes the recording material and study site in detail. The algorithm underlying the periodicity-coded non-negative matric factorisation has been published previously (Lin et al. 2017), and all the Matlab code used in this study is available from Code Ocean. However, some details in the analyses are missing.*

*Line 409, Figure 8: I would suggest changing the numbering of the spectral features of each source, or splitting spectral features by source to better see which temporal activations/periodicities correspond to which source.* ***DONE.***

**REPLY:** Following the review’s suggestion, we separated the visualization of spectral features, temporal activations, and periodicity patterns according to different sound sources.

**COMMENT:** *Line 416: Why 90 spectral features? Was it an arbitrary choice or was it justified?* ***DONE.***

**REPLY:** The number of spectral features applied in this study is an arbitrary decision. A simulation showed that PC-NMF performed relatively worse when fewer than 60 spectral features were used [39]. Therefore, we expected a larger dictionary to retain the acoustic diversity of real-world data. (**Lines 334-338**)

**COMMENT:** *Line 428: Similarly, why 15 minutes and 0.5? Did you optimise the parameters?* ***DONE.***

**REPLY:** The choice of number of frames is mainly due to the limitation of VRAM of our GPU. We have discussed this part in **Lines 471 and 530-534**. The choice of sparsity depends on the characteristics of sound sources. Most of soundscapes have components that are either narrowband or broadbands, therefore we used a sparsity of 0.5 in this study. The choice of sparsity was discussed in **Lines 328-334**.

**COMMENT:** *Line 434-435: How did the first author determine whether a spectral feature was mislabelled?* ***DONE.***

**REPLY:** This is done by searching spectral features activated simultaneously in randomly selected audio events (e.g., shipping sounds) but recognized as different sound sources by the PC-NMF. (**Lines 554-557**)

**COMMENT:** *Did you use absolute LTSA levels to compare the sound levels of the different sources?* ***DONE.***

**REPLY:** In this study, we didn’t compare the sound levels between different sound sources. We only measured the integrated energy levels to evaluate the phenology patterns. Integrated energy levels of each separated sound source were measured from the reconstructed LTSA. Because the reconstructed LTSA was linearly separated from a prewhitened LTSA, the values only approximately represented the signal strength relative to the background noise. Therefore, the integrated energy levels were normalized to a range between 0 and 1 by the minimum and maximum values to visualize the temporal variations of each sound source. (**Lines 574-579**)

**COMMENT:** *Which criteria did you use in the time-lagged correlation analysis to define a recurring pattern?* ***DONE.***

**REPLY:** The relative intensities were applied to the measurement of the time-lagged correlations to investigate the recurring patterns. The local maximum of correlation scores within 24 h was selected to examine the diurnal pattern. We measured the correlation envelope for the lunar and seasonal cycles by using a 1-day sliding window and identified the local maximums. (**Lines 579-583**)

**RESPONSE TO REVIEWER 3**

**COMMENT:** *In this manuscript the authors present a study on the northeastern Taiwan soundscape using PC-NMF, a method for source separation proposed by the authors in 2017. They claim that “audio source separation will play a crucial role in soundscape-based ecosystem monitoring and will facilitate the assessment of acoustic diversity that is challenging in noisy environments“ and aim to provide a successful example of this in the proposed manuscript. Even though the motivation to use source separation for marine soundscapes is clear from the text, the main contribution and innovation is not.*

*The authors provide references to the chosen source separation method but fail to motivate the reason why source separation is needed in this case, why is such a method the best option for this study amongst the other state-of-the-art methods in audio source separation, and most importantly, they fail to expose the main findings of this study leaving the reader unsure on the contribution and innovation of this manuscript, specially taking into account that PC-NMF, its motivation and applications have already been discussed in the literature.* ***DONE.***

**REPLY:** We appreciate the constructive suggestions from the reviewer. In the revised manuscript, we discussed that most of bioacoustics studies developed detectors and noise filters. However, parameter tuning is labor-intensive and often needs to repeat when analyzing data collected from a new environment. Although the recent developments in deep learning have reduced the difficulty in classifying animal vocalizations and human speech [29–32], most of the techniques require a large number of preexisting labels for supervised training (**Lines 103-107**). The application of PC-NMF can greatly reduce the labor-intensive nature of the works and the need of prior knowledge, especially for acoustic data that displayed apparent diversity in phenological patterns (section of **Applying Audio Source Separation in Assessing Soundscapes**). Performing clustering on the source separation results also enabled a more comprehensive assessment of acoustic diversity and revealed the difference in spectral characteristics and phenological patterns between groups of marine soniferous animals. As ocean noise continues to increase, the use of audio source separation to monitor marine soundscapes and investigate spatiotemporal changes in biophony, anthropophony, and geophony can facilitate the tracking of biodiversity changes on both regional and global scales. (section of **Retrieving Ecological Information from Soundscapes**).

**COMMENT:** *Overall I believe the study needs to be revisited, the methodology reconsidered and the text improved to make sure to include:*

*- literature review on other source separation techniques that could be used*

*- The motivation and justification for the chosen source separation method regarding this data*

*- Clear explanation and steps of the methodology used*

*- Justification for the technical choices made*

*- Statement of the current (before the study) knowledge of the northeastern Taiwan soundscape*

*- Clear description of the findings of their study and their impact*

***DONE.***

**REPLY:** The revised manuscript has added a section to review the current knowledge on the marine soundscapes off northeastern Taiwan (section of **Marine Soundscapes off Northeastern Taiwan**). We discussed the advantage of using PC-NMF over the other methods, including the capability of unsupervised feature learning (**Fig 8** in the revised manuscript) and the flexibility to develop a model in a semi-supervised approach (section of **Applying Audio Source Separation in Assessing Soundscapes**). We also added more paragraphs to demonstrate how applied techniques can improve acoustic diversity assessment and reveal the difference in spectral characteristics and phenological patterns between groups of marine soniferous animals (**Fig 7** in the revised manuscript). We discussed how the result of soundscape information retrieval could improve our understanding of the community of marine soniferous animals in a continental shelf environment and their interactions with other species and anthropogenic activities (section of **Retrieving Ecological Information from Soundscapes**). We have added more details on the methodology used, especially the Sections of **Audio Source Separation**, **Analysis of Acoustic Phenology**, and **Evaluation of Acoustic Diversity**. We believe that we have made the appropriate revisions according to the review comments.

**COMMENT:** *Regarding source separation: From the text, the authors seem to have some confusion over the terms “simultaneously” and “overlapping”, which unfortunately does not help motivate the need for advanced source separation techniques in this study. An example of this confusion can be found at line 109 where the authors wrongly state that “signals recorded simultaneously cannot be separated on the basis of clustering” as one can imagine a mixture of a low and a high pitched source to be separated through “clustering” as long as their overlap remains in time and not frequency. Other examples can be found at line 110 and 120.*

*If two sounds happen simultaneously they overlap in time but it does not mean that they also overlap in frequency. As a matter of fact, in source separation we often use time-frequency representations to exploit this: less overlap between sources in the frequency domain allows for simpler techniques to separate sources. Of course if the sources do not overlap in time, then the separation can simply be done in the time domain.* ***DONE.***

**REPLY:** We have revised the text as “Low audio quality owing to the simultaneous recording of multiple sound sources increases clustering uncertainty.” to prevent confusion. Indeed, clustering algorithms (such as GMM) may separate a low-pitch sound and a high-pitch sound based on the posterior probabilities. Despite that, most clustering techniques were designed to deliver categorial information instead of reconstructing the independent sources from a mixture. The dataset used in this study contains multiple types of biotic and abiotic sound sources that may be overlapping in time or frequency domains (**Fig 2 & 3** in the revised manuscript), and each sound source also possesses spectral variability. We demonstrated that the applied technique of audio source separation not only reduces the interference among sound sources but also enables further applications, such as the variability assessment of source behaviors (**Fig 4 & 7** in the revised manuscript). Most importantly, the applied technique can transform single-channel audio into multiple channels that encode the dynamics of biophony, geophony, and anthropophony. The outcomes will expand the deliverables of soundscape monitoring that are difficult to achieve by using conventional approaches.

**COMMENT:** *In addition, the different sound properties of sources also play a key role when deciding which source separation method to use. For example stationary sources can easily be separated and removed through Wiener filtering. Therefore the use of NMF to remove electrical noise (visibly stationary in Figure 1 and 2) is unjustified.*

*As a summary, here are some questions to take into consideration (and discuss) when deciding which method to use in source separation:*

*- Do sources overlap in time?*

*—> No : simple separation in time domain*

*-> Yes : Do they overlap in frequency?*

*-> No : simple separation in time-frequency domain*

*-> Yes : Do the sources share properties (e.g. stationary, impulsive, broadband, harmonic … )?*

*-> No : basic modelling and filtering*

*-> Yes : advanced source separation techniques such as NMF*

***DONE.***

**REPLY:** We appreciate the constructive suggestions from the reviewer. In the revised manuscript, we have added more details to describe the frequency characteristics and variability for each sound source (**Lines 206-214**). The suggested data processing procedures highly depend on the prior knowledge on each separated sound source (including biotic and abiotic sounds). It requires a series of modeling and noise filtering, which may be difficult for managers and stakeholders interested in using soundscapes in ecosystem monitoring and conservation management. By contrast, the audio source separation and clustering techniques applied here significantly reduced the labor-intensive nature of the works.

**COMMENT:** *The recordings in this study can be divided into four sources: abiotic sound, fish chorus, cetacean vocalisations and electrical noise. As the electrical noise is stationary it should be removed through filtering in an initial pre-processing stage, leaving abiotic, fish and cetaceans as sources to be considered further. From these three sources, we could safely assume the abiotic sounds to have considerably distinct properties to the biological sources, making the separation between fish and cetaceans the real technical challenge in this study. However, from the text we find out that these two types of biological sounds do not always overlap in time (e.g. Line 179) and furthermore, they seem to only overlap in frequency between 1 and 3kHz (line 135 onwards), raising questions on the real need for advanced source separation methods such as PC-NMF. The authors did not provide any discussion on this, nor strong arguments for the use of PC-NMF in this study.* ***DONE.***

**REPLY:** The electrical noise in the MACHO recordings is not stationary. Electrical noise consisted of spectral peaks distributed in various frequencies and displayed a highly unpredictable temporally changing pattern. A few noise-related spectral peaks were more predictable in a day, but their frequencies also changed between months. (**Lines 211-214**). In addition, abiotic sounds may extend to 20–30 kHz and often recorded in high intensities (especially for shipping noise and sounder signals). Therefore, abiotic sounds can mask low-frequency whistles of delphinids and fish choruses. If we applied conventional approaches, such as bandpass filters, the detection results would include lots of false alarms (see **Supplementary Figure 1**).

The application of PC-NMF has two advantages: The first advantage is the unsupervised feature learning and the automatic recognition of source indicators according to the assumption of source-specific periodicity. This part was discussed in **Lines 327-348**. The second advantage is the visualization of model parameters, which allow experienced users to change the source indicators of spectral features and subsequently improve the unsupervised learning result. This part was discussed in **Lines 355-366**. The application of PC-NMF can greatly reduce the labor-intensive nature of the works and the need of prior knowledge, especially for acoustic data that displayed apparent diversity in phenological patterns (section of **Applying Audio Source Separation in Assessing Soundscapes**). Performing clustering on the source separation results also enabled a more comprehensive assessment of acoustic diversity and revealed the difference in spectral characteristics and phenological patterns between groups of marine soniferous animals. As ocean noise continues to increase, the use of audio source separation to monitor marine soundscapes and investigate spatiotemporal changes in biophony, anthropophony, and geophony can facilitate the tracking of biodiversity changes on both regional and global scales. (section of **Retrieving Ecological Information from Soundscapes**).

**COMMENT:** *Line 51: “This study applied machine learning techniques to analyze underwater recordings transmitted from a deep seafloor observatory.”: what are the other machine learning techniques used in this study? Be aware NMF is not always considered machine learning in the literature, specially on its basic form like in PC-NMF.* ***NO CHANGE.***

**REPLY:** The applied machine learning techniques in this study, including NMF and k-means clustering, are not comparable to modern techniques based on deep learning. Despite that, they do have a learning object in the algorithm. For NMF as an example, it can learn a set of spectral features and temporal activations that are initialized by random values and reconstruct a spectrogram by iteratively updating the parameters in an unsupervised manner (or self-supervised approach). If we search the term NMF in the Journal of Machine Learning Research, one can easily find many articles discussing its applications. Although the most basic form of NMF has been developed for more than 20 years (Lee & Seung 1999. Nature 401, 788–791.), it has been widely applied in music and speech separation (but rarely applied in bioacoustics and ecoacoustics). Even for the state-of-the-art speech separation models based on deep learning, these models were trained to map noisy recordings to clean speech, but a database of clean sounds is unlikely to obtain in marine environments.

**COMMENT:** *Line 139: “Despite the significant frequency overlap among sound sources…” —> 2kHz overlap is generally not considered significant, therefore if it is significant in this case please explain why.* ***DONE.***

**REPLY:** The frequency range of fish chorus is entirely within the frequency range of abiotic sounds (**Figure 3** in the revised manuscript). In addition, cetacean vocalizations also partially overlap with abiotic sounds (mainly in the frequencies < 30 kHz). To prevent confusion, we removed the text in the revised manuscript.

**COMMENT:** *Line 190: “By applying a threshold of 0.05..“: Provide justification.* ***DONE.***

**REPLY:** The applied threshold ensured that the false alarm rates of the detected fish chorus and cetacean vocalizations were ≤ 5% (**Line 260**)

**COMMENT:** *My understanding of PC-NMF (the text was not clear on this so I went and read reference 33): First apply standard NMF approximating a time-frequency representation of the sound recording into a multiplication of two non-negative matrices, one known as the “dictionary” containing the spectral basis and another known as the activation matrix telling us when and how those spectral basis are present in the signal. Now the goal becomes to determine which spectral basis belong to the target source we want to separate, in the case of underwater recordings, the biological sounds. In PC-NMF they assume the biological sounds to be periodic unlike the interfering anthropogenic/environmental sounds, and so they analyse the repetitions of patterns in the activation matrix by taking the FFT of every row in the activation matrix. Every spectral template (i.e. row in activation matrix) that peaks at the same time in the FFT is considered to be originated from the same biological source. In this way one can group spectral basis into different biological sources and noise to continue with the standard separation in NMF.*

*Therefore the interesting questions about PC-NMF are :*

*-> Does the main assumption of biological sound periodicity always hold? One can imagine boats also producing period sounds, how does this affect PC-NMF? How important is the difference in period between sound sources?*

***DONE.***

**REPLY:** Examples of reoccurring behavior of biological sounds can be found in the second paragraph of the introduction (**Lines 75-86**). In addition, previous investigations on the MACHO recordings also revealed that delphinid whistles and one type of fish chorus displayed evident periodical behaviors.

The main assumption of the PC-NMF is the “source-specific periodicity”. The first article of PC-NMF demonstrated the feasibility of separating biological chorus and other noise source because biological sounds generally display more evident diurnal periodicity than non-biological sounds. By assuming source-specific periodicity, we expect the PC-NMF should work for acoustic data that display apparent diversity in phenological patterns. Although the recurring behavior of wildlife varies based on various biotic and abiotic factors, the PC-NMF may still help us examine the evolution of acoustic phenology in response to a changing environment. (**Lines 317-326**).

As we discussed in the revised manuscript, the PC-NMF may not be able to solve a complicated dataset (**Lines 362**). Therefore, this study also showed the procedure to manually correct the source indicators learned by the PC-NMF and improved the separation performance (**Lines 196-200**). The flexibility to develop a model in a semi-supervised manner is crucial when we don’t have sufficient preexisting labels to train a model using supervised learning. This was further discussed in **Lines 358-366**.

**COMMENT:** *How many spectral basis (i.e. rank) should one pick to make sure there is no confusion between sources?* ***DONE.***

**REPLY:** The number of spectral features applied in this study is an arbitrary decision. A simulation showed that PC-NMF performed relatively worse when fewer than 60 spectral features were used [39]. Therefore, we expected a larger dictionary to retain the acoustic diversity of real-world data. (**Lines 334-338**)

**COMMENT:** *What is the a priori knowledge needed to be able to allocate spectral basis to certain species (i.e. sound sources)? Knowing how to cluster spectral templates does not imply knowing its sound source. None of the above were discussed in the presented text.* ***DONE.***

**REPLY:** The prior knowledge for the applied technique of audio source separation is the number of sound sources we want to separate. In the revised manuscript, we added a section to describe the current knowledge regarding the marine soundscapes off northeastern Taiwan (**Lines 148-182**), which represents the baseline information for us to determine the number of separated sound sources. We agree with the review’s comment because the PC-NMF may not separate two sound sources due to the uncertainty of periodicity. Therefore, we demonstrated the procedure to manually correct the source indicators learned by the PC-NMF and improve the separation performance (**Lines 196-200**). The flexibility to develop a model in a semi-supervised manner is crucial when we don’t have sufficient preexisting labels to train a model using supervised learning. This was further discussed in **Lines 358-366**.

**COMMENT:** *“two layers of non-negative matrix factorisation”: PC-NMF seems to perform only one NMF optimisation, and therefore has only “one layer”. A second layer of NMF could for example be taking the learnt templates of a source and fixing them on another semi-supervised optimisation round —> does this happen here?* ***DONE.***

**REPLY:** In the revised manuscript, we have clarified the procedures of applying PC-NMF to perform audio source separation of the MACHO recordings at **Lines 544-572**. Two NMF layers were used in the training phase (one for feature learning and another for learning source indicators). However, in the prediction phase, only the feature learning layer was used. We have revised the text in the abstract as “A source separation model based on the periodicity-coded nonnegative matrix factorization was developed to perform unsupervised feature learning. The model learned to discriminate groups of spectral features using the assumption of source-specific periodic patterns with minimal supervision and prove to be effective in the separation of sounds made by cetaceans, soniferous fish, and abiotic sources from the deep-water soundscapes off northeastern Taiwan.” (**Lines 27-32**).

**COMMENT:** *Line 52: Which training?* ***DONE.***

**REPLY:** The training here refers to the training phase of source separation procedures (using a subset of LTSA to learn 90 spectral features without preexisting labels). We have clarified the procedures of audio source separation in the revised manuscript at **Lines 544-572**. In addition, we revised the text in the author summary as “In particular, our computational approach does not require preexisting labels in the procedure of model building.” (**Line 52**)

**COMMENT:** *Line 130 onwards: How did you decomposed the LTSA into four independed sound sources? What a priori knowledge did you use? This should be discussed in depth.* ***DONE.***

**REPLY:** The detailed procedures regarding the decomposition of an LTSA can be found in **Lines 544-572**. The prior knowledge for the applied technique of audio source separation is the number of sound sources we want to separate. In the revised manuscript, we added a section to describe the current knowledge regarding the marine soundscapes off northeastern Taiwan (**Lines 148-182**), which represents the baseline information for us to determine the number of separated sound sources.

**COMMENT:** *Line 159: “Compared to the pattern observed in abiotic sounds, the patterns of the two biophonic sound sources showed clear periodic structures.” — > Where can we see these clear periodic structures?* ***DONE.***

**REPLY:** We have changed the text as “The time-lagged correlations indicated that abiotic sounds, cetacean vocalizations, and fish choruses displayed different periodic patterns (Fig 5).” (**Line 228**).

**COMMENT:** *Line 192 onwards: How did the clustering work? What a priori information was used? There should be a major focus on this as it is where the contribution of this paper could rely.* ***DONE.***

**REPLY:** The revised manuscript added more details on the procedure of clustering analysis (**Lines 592-622**). We did not use any prior knowledge for the clustering to determine the number of clusters (*k*). Instead, we measured the ratio between the mean within-cluster variation and the overall variation for different *k* values. The relationship between the number of clusters and the explained data dispersion can be found in Supplementary Figure 2. (**Lines 612-622**).

**COMMENT:** *Line 241: “… because most of the biological activities occur periodically. “ —> Please provide justification or reference or this claim.* ***DONE.***

**REPLY:** Examples can be found in the second paragraph of the introduction (**Lines 75-86**). In addition, previous investigations on the MACHO recordings also revealed that delphinid whistles and one type of fish chorus displayed evident periodical behaviors. In the revised manuscript, we removed the text “because most of the biological activities occur periodically” to prevent confusion.

**COMMENT:** *Paragraph starting at Line 246: How was the process manually modified to increase its performance? Which were the spectral basis in correctly assign? And most importantly, why?* ***DONE.***

**REPLY:** In the revised manuscript, we compare the separation performance before and after the manual correction of source indicators: “The source separation model correctly identified 67.33% of cetacean vocalizations when the false-positive rate was set to 5%, but it failed to separate fish choruses and low-frequency abiotic sounds. We investigated the temporal activations and manually adjusted the source indicators of the two spectral features associated with fish choruses. After the adjustment, the model outperformed conventionally applied band-pass filters (S1 Fig.) and correctly identified 73.10% of fish choruses and 85.99% of cetacean vocalizations at the same false-positive rate (5%).” (**Lines 194-200**).

We also explained the procedure of manual modification of source indicators in **Lines 554-559**. In addition, we discussed the necessity of this semi-supervised approach in **Lines 358-366**. “As demonstrated in this study, the source separation performance improved when the source indicators learned by the second NMF layer were manually corrected. This can be done by inspecting the learned temporal activations of the first NMF layer to determine whether a source indicator is appropriate. In some cases, manually adjusting model parameters are necessary because our assumption of source-specific periodicity may not be able to solve a complicated dataset. The flexibility to develop a model in a semi-supervised manner allows users to combine models trained from different datasets and manipulate their source indicators to improve the generalizability of audio source separation.”

**COMMENT:** *Line 333 : I’m confused, which training data do you refer to here?* ***DONE.***

**REPLY:** The training data refer to the 12-days subsampled dataset for model training. The text has been changed as “A GPU with massive VRAM allows us to use more data to train an NMF-based source separation model and increase the amount of time-dependent information learned by each spectral feature.” (**Line 471**)

**COMMENT:** *Line 416 : Justify the choice of rank 90 or at least provide a discussion on the rank choice impact.* ***DONE.***

**REPLY:** The number of spectral features applied in this study is an arbitrary decision. A simulation showed that PC-NMF performed relatively worse when fewer than 60 spectral features were used [39]. Therefore, we expected a larger dictionary to retain the acoustic diversity of real-world data. (**Lines 334-338**)

**COMMENT:** *Paragraph starting at line 464 is not clear, more detail on how to perform the clustering is key for other researchers trying this method to find sources.* ***DONE.***

**REPLY:** The revised manuscript added more details on the method of clustering in order to increase the reproducibility (section of **Evaluation of Acoustic Diversity**).

**COMMENT:** *Possible contributions:*

*- Abstract: Identification of diurnal and seasonal interactions between cetaceans and soniferous fish.*

*- Line 55: Finding: continental shelf environment has a high diversity of sound-producing animals, and the community structure changes with daily, lunar, and seasonal cycles.* ***NO CHANGE.***

**COMMENT:** *- Line 58: “A database of anthropogenic noise has also been established and used to evaluate how human activities may affect the behavior of sound-producing animals.” —> Did not find any other reference to this in the text.* ***DONE.***

**REPLY:** We rewrote the sentence as “The techniques applied also generated a database of environmental and anthropogenic sounds, which can facilitate future investigations on how human activities affect the acoustic environment and biodiversity.” (**Line 58**)

**COMMENT:** *In order to better understand if these are contributions background is needed. What did we know before this study? It is unclear what information is known and what is found through the presented analysis, and its impact. How do these findings advance the field?* ***DONE.***

**REPLY:** The revised manuscript added a section to describe the current knowledge regarding the marine soundscapes off northeastern Taiwan. We discussed the advantage of using PC-NMF over the other methods, including the capability of unsupervised feature learning (**Fig 8** in the revised manuscript) and the flexibility to develop a model in a semi-supervised approach (section of **Applying Audio Source Separation in Assessing Soundscapes**). We also added more paragraphs to demonstrate how applied techniques can improve acoustic diversity assessment and reveal the difference in spectral characteristics and phenological patterns between groups of marine soniferous animals (**Fig 7** in the revised manuscript). Furthermore, we discussed how the result of soundscape information retrieval could improve our understanding of the community of marine soniferous animals in a continental shelf environment and their interactions with other species and anthropogenic activities (section of **Retrieving Ecological Information from Soundscapes**).

**COMMENT:** *Line 156: “We noticed that audio data were heavily corrupted … Therefore, these data were not included in the subsequent analyses.” —> Please provide further explanation for this as unjustified partial use of data is not scientifically acceptable.* ***DONE.***

**REPLY:** We added explanations for this part in **Lines 223-228**. “The audio data from March 24 to July 1, 2013, recorded high-intensity broadband noise in frequencies ≥ 30 kHz, leading to incorrect detection of cetacean vocalizations. Because the high-frequency range was too heavily corrupted to identify echolocation clicks manually, the intensities of cetacean vocalizations were manually set to 0 to prevent bias in the phenological pattern analysis.” Because most of the detections of cetacean vocalizations during this period were high-intensity broadband noise, the inclusion of this set of data will bias the analysis result of phenological patterns. The current approach is the same as the manual removal of false alarms, widely used in bioacoustics studies.

**COMMENT:** *Line 214 onwards: The cetacean clustering and analysis should be discussed in depth alongside some data representation supporting the claims such as the one on Line 218.* ***DONE.***

**REPLY:** Following the review’s suggestion, we revised the **Fig 7** to visualize the spectral characteristics and temporal variations (diurnal and seasonal changes).

**COMMENT:** *Line 269: Which type of fish? How did you identified it? Where is the report on their spectral, diurnal and seasonal patterns?* ***DONE.***

**REPLY:** At this moment, we are unable to identify the species responsible for the two types of fish choruses because the ground truth is not available. However, Myctophidae fishes in Perth Canyon [52] were discovered to display similar chorusing behavior. The discussion has been added in **Line 377**.

**COMMENT:** *Line 294: “According to previous experiments, …“ —> which ones?* ***DONE.***

**REPLY:** We have revised the text as “While delphinids can acquire acoustic information regarding the identity, abundance, and location of their prey through passive listening [54,55], soniferous fish may also suppress their calling behavior when they detect the sounds of their predators [56].” (**Line 413**)

**COMMENT:** *Paragraph starting at 432: Does this mean that the results presented were issued from a supervised approach? If manually labelled data was used to ‘learn’ spectral basis of the sources through an initial NMF and were then fixed for a semi-supervised NMF round in the rest of the data, please make sure to state earlier in the text section that the results are not issued from an unsupervised method as is currently claimed. Example: how does the statement at line 122 “First, we applied audio visualization and source separation techniques to demonstrate the feasibility of separating biotic and abiotic sounds in an unsupervised manner” with the statement at line 439 “The analysis of the entire dataset was conducted in a supervised manner”?* ***DONE.***

**REPLY:** We have clarified the procedure of audio source separation in the revised manuscript at (**Lines 544-572)**. “We divided the source separation into a training phase, a prediction phase, and a reconstruction phase. The training phase was to learn the source-specific spectral features from a small subset of MACHO recordings. The prediction phase was to learn the temporal activations of the source-specific spectral features from the entire dataset of MACHO recordings. After the prediction phase, each sound source was reconstructed using the source-specific spectral features and the associated temporal activations.

In the training phase, we selected the audio data of the first day of each month from November 2011 to October 2012 (34,541 audio clips) to generate a prewhitened LTSA. In the training phase, the unlabeled LTSA was consumed by the PC-NMF to obtain 90 spectral features and their source indicators. The source indicators were reviewed by the first author to check whether any spectral features were mislabeled by searching spectral features activated simultaneously in randomly selected audio events (e.g., shipping sounds), but recognized as different sound sources by PC-NMF. Following manual inspection, only spectral features and source indicators were saved as the prior knowledge for the prediction and reconstruction phases. After the training phase, the first author manually annotated fish choruses and cetacean vocalizations from the subsampled dataset to measure the true-positive rate and false-positive rate of the source separation model.

In the prediction phase, we only used the first NMF layer because the 90 spectral features and their source indicators were fixed. We computed a prewhitened LTSA for the audio recordings of each day and applied NMF to learn the temporal activations of the 90 spectral features. The temporal activations were initialized by random values and updated for 200 iterations.”

**COMMENT:** *Line 461: Justification missing for the 10 and 1 min choices.* ***DONE.***

**REPLY:** The revised manuscript has explained the choice of 10 and 1 min: “The applied criteria enabled us to ensure that the identification of an acoustic event was not influenced by isolated or faint signals, thus increasing the confidence of diversity assessment.” (**Line 596**)

**RESPONSE TO REVIEWER 4**

**COMMENT:** *In general it is a well-written article. Methodologically, I would suggest that you be more detailed about the technique used, especially if you want to attract reader engineers who want to contribute to the algorithms.* ***DONE.***

**REPLY:** We appreciate the constructive suggestions from the reviewer. We have added more details on the methodology used, especially the Sections of **Audio Source Separation**, **Analysis of Acoustic Phenology**, and **Evaluation of Acoustic Diversity**. We believe that we have made the appropriate revisions according to the review comments.
