# Supplementary material for: Sensing ecosystem dynamics via audio source separation: A case study of marine soundscapes off northeastern Taiwan
Source: PLoS Comput Biol. 2021 Feb 18;17(2):e1008698. doi: 10.1371/journal.pcbi.1008698 (PMC7891715; doi:10.1371/journal.pcbi.1008698)
Supplement: Attachment — Submitted filename: review.docx [file pcbi.1008698.s005.docx]

The authors have made huge improvements to the manuscript. They added sections and paragraphs to provide the information missing from the previous version. They included many details to clarify their claims where it was needed. The readability and flow of the revised manuscript has substantially improved.

The responses and explanations to my previous comments are clear. I only have a few remaining questions regarding the new contents of the manuscript.

**Introduction**

The revised introduction provides a more furnished overview of the previous literature and summary of available methods, and adds a description of the prior knowledge about the study environment.

Notes about the changes:

- The description of conventional bioacoustics approaches and their caveats is now more detailed.
- Blind source separation (BSS) and the added value of this approach now benefit from a clear introduction, and the authors provide some examples.
- The assumptions and principles of the periodicity-coded non-negative matrix factorisation (PC-NMF) are now clearly presented.
- The authors presented the prior knowledge about the marine soundscape in the study area (i.e. known sound sources and their frequency ranges).
- The value of the MACHO recordings and the challenges that their analysis represent were highlighted in the final paragraph of the revised introduction.

Remarks:

- Line 123-124: The sentence *“the low-rank assumption cannot handle highly dynamic soundscapes”* is not clear*.*

**Results**

The authors separated the Results in three subsections: *source separation performance*, *acoustic phenology and temporal interactions*, and *diversity of biotic and abiotic sounds*. It is now easier to follow the train of thought of the authors. They also added many details in the description of their results, including some reminders of the Methods, which further enhance readability and understanding.

Notes about the changes:

- The authors justified the use of 12 days to compute long-term spectral average and to learn spectral features with computational (memory) costs.
- The authors presented the initial results of source separation, before explaining how they manually adjusted the source indicators. The improvement of the source separation performance by this semi-supervised procedure is now clearer.
- The added supplementary Figure S1 provides a clear comparison of PC-NMF and conventional band-pass filter-based detectors.
- The authors described the MACHO electrical noise in more details, which clarifies why simple noise-removal techniques would not work in this case.
- The description of the temporal correlations between fish choruses and cetacean sounds has been reworded and is now clearer.
- The authors explained what the threshold of 0.05 on relative intensities corresponds to in terms of detection performance.
- The authors reminded the readers that they used k-means clustering before describing the different clusters. This reminder helps to understand this section of the Results.
- The description of the clusters detected for each sound source (abiotic sounds, fish choruses, and cetacean sounds), their spectral characteristics, and their temporal variation patterns is more detailed in the revised manuscript.
- The panels in the revised Figure 7 now display each cluster separately. Figure 7 is now much larger, but its information content and readability greatly improved.

Remarks:

- The authors detailed the nature of the data corruption and explained that they set the relative intensity of cetacean sounds to 0 over the corrupted period. I still wonder how this artificial gap in cetacean sounds is affecting the results of the time-lagged correlation analysis.
- The authors used the term *“temporal interactions”* throughout the Results. There is no indication of the mechanisms underlying these observations, therefore I feel like *“temporal correlations”* would be a more adequate term.

**Discussion**

The authors developed the paragraphs of the discussion that were too succinct, and added some discussion of important ideas or notions that were missing in the previous version of the manuscript. The Discussion now provides a more critical summary of the results, and has a deeper perspective about the future perspectives of the PC-NMF method.

Notes about the changes:

- The authors detailed possible applications of audio source separation and its advantages over other methods. They discussed the validity of the PC-NMF assumption that sound sources have specific periodicity patterns.
- The authors now discuss the performance of PC-NMF. They explain that it depends on sparsity and feature number and give a concrete meaning to these parameters.
- The flexibility allowed by the semi-supervised adjustment of source activations is now discussed as an asset for generalisation.
- The authors compared the dictionary of spectral features learnt by the PC-NMF with their prior knowledge of the sound sources forming the soundscape off northeastern Taiwan.
- Sound sources are now separate in Figure 8. The readability of this figure is improved.
- The authors made a much welcome effort to identify the species which could have produced the fish choruses and cetaceans sounds detected in the MACHO recording. They noted the similarity between one of the fish choruses and the sounds of Myctophids. They listed common cetacean species off Taiwan (delphinids and sperm whales), but did not go further in the identification of cetacean species corresponding to the six clusters of cetacean sounds. This addition is a great improvement of the manuscript.
- The authors provided an example of how acoustic diversity could be used to study community structure and behaviour: the intense night-time foraging of cetaceans could be used to define essential ecological areas in the study region.
- The authors described in more details the importance of anthropogenic sounds for marine conservation. They explained that abiotic sound sources could be used to study the impacts of human activities on marine ecosystems: for instance, the temporal variations of echosounder-related clusters could provide information about fishing effort in the area. This section gives more weight to the concerns raised by the ongoing increase of anthropogenic sounds.
- The authors provided additional details about the computational constraints of the PC-NMF methods, which were mostly related to VRAM limitations. They discussed the feasibility and outcomes of using simpler or more complex models.

**Methods**

The authors added many precisions to the Methods section. They also clarified many points that were confusing or only touched on in the previous version. The improved Methods section would make it easier to reproduce the analysis, and helps understanding the rest of the manuscript.

Notes about the changes:

- The authors corrected their statement about data availability.
- The authors developed their description of the three phases of the PC-NMF: training, prediction, and reconstruction. It is now clearer what the two layers of the PC-NMF correspond to, and what their input and output is.
- The authors mentioned that both layers are fit through 200 iterations of reconstruction error minimisation.
- The authors explained that they detected and corrected source misidentifications by finding similar events which were not assigned to the same source.
- They precised that they manually annotated a subset of data to calculate false positive rates.
- Equations 5 and 6 are important additions to understand the source identification and reconstruction steps.
- The authors detailed how they identified local maxima of time-lagged correlation to define diurnal, lunar, and seasonal patterns within and between sound sources.

Remarks:

- The authors explained that they measured clustering quality as the ratio between within-cluster and dataset dispersions (R values). However, R values are not used in the manuscript (except possibly in Figure S2, but this isn’t clear).
- The authors claimed in the Methods that they selected both a small and a large number of clusters after the k-means clustering. However, only one cluster number is mentioned in the Results.
