# Supplementary material for: Sensing ecosystem dynamics via audio source separation: A case study of marine soundscapes off northeastern Taiwan
Source: PLoS Comput Biol. 2021 Feb 18;17(2):e1008698. doi: 10.1371/journal.pcbi.1008698 (PMC7891715; doi:10.1371/journal.pcbi.1008698)
Supplement: Attachment — Submitted filename: ResponseLetter.docx [file pcbi.1008698.s006.docx]

**RESPONSE TO GUEST EDITOR**

**COMMENT:** *The abstract should not claim "A source separation model ... was developed" since the source separation model is one developed in prior work.* **Done.**

**REPLY:** Following the editor’s suggestion, the sentence has been revised as “A source separation model based on the assumption of source-specific periodicity was used to factorize time-frequency representations of long-duration underwater recordings.” (**Line 27**)

**COMMENT:** *"minimal supervision" is not the same as "unsupervised". Authors must be careful, and not claim a method is "unsupervised" when the procedure involves manual interventions to achieve the desired per-class outputs. It can be correct to label a component, for example a single pass of PC-NMF, as an unsupervised process. However, the current wording in the abstract is imprecise, and could mislead a reader into believing the overall procedure is unsupervised.* **Done.**

**REPLY:** We appreciate the constructive suggestions from the guest editor and the reviewers. The previous version mentioned our method as “unsupervised feature learning” in the abstract. To prevent confusion, we have removed the term “unsupervised” and rewrote this section as: “A source separation model based on the assumption of source-specific periodicity was used to factorize time-frequency representations of long-duration underwater recordings. With minimal supervision, the model learned to discriminate source-specific spectral features and prove to be effective in the separation of sounds made by cetaceans, soniferous fish, and abiotic sources from the deep-water soundscapes off northeastern Taiwan.” (**Line 27-32**)

**COMMENT:** *The definition of "A BSS model aims..." needs to be made technically correct.* **Done.**

**REPLY:** The definition of BSS has been corrected as “A BSS model aims to separate a set of sound sources from a mixture, without prior information specific to individual sources or the mixing process.” (**Line 120**)

**RESPONSE TO REVIEWER 2**

**COMMENT:** *Line 123-124: The sentence “the low-rank assumption cannot handle highly dynamic soundscapes” is not clear.* **Done.**

**REPLY:** We rewrote the description associated with RPCA as “Robust principal component analysis (RPCA) has been successfully applied to separate accompaniment and singing voice by assuming the accompaniment to be in a low-rank subspace and the singing voice to be relatively sparse [36]. When such low-rank assumption does not hold (e.g., when biotic and abiotic sounds are highly dynamic), the RPCA cannot yield satisfactory performance.” (**Line 123-128**)

**COMMENT:** *The authors detailed the nature of the data corruption and explained that they set the relative intensity of cetacean sounds to 0 over the corrupted period. I still wonder how this artificial gap in cetacean sounds is affecting the results of the time-lagged correlation analysis.* **Done.**

**REPLY:** We compared the results of time-lagged correlation analysis based on the original data and the manually corrected data. We didn’t observe any differences in the peak correlation values. However, the correlation values within at the intervals less than 100 days were inflated because of the false positive detections of cetacean sounds. We provided more details about this part in **Lines 229-232**: “The audio data from March 24 to July 1, 2013, recorded high-intensity broadband noise in frequencies ≥ 30 kHz, leading to incorrect detection of cetacean vocalizations and adding noise in the analysis of time-lagged correlations.”

**COMMENT:** *The authors used the term “temporal interactions” throughout the Results. There is no indication of the mechanisms underlying these observations, therefore I feel like “temporal correlations” would be a more adequate term.* **Done.**

**REPLY:** Following the review’s suggestion, we have changed the term “temporal interactions” to “temporal correlations” in the revised manuscript.

**COMMENT:** *The authors explained that they measured clustering quality as the ratio between within-cluster and dataset dispersions (R values). However, R values are not used in the manuscript (except possibly in Figure S2, but this isn’t clear).* **Done.**

**REPLY:** The revised manuscript has added R values in the section associated with the clustering results of fish chorus and cetacean vocalizations **(Line 286)**. We also explained how we use the R values to find an optimal number of clusters **(Lines 626-632)**.

**COMMENT:** *The authors claimed in the Methods that they selected both a small and a large number of clusters after the k-means clustering. However, only one cluster number is mentioned in the Results.* **Done.**

**REPLY:** This study only selected one cluster number for each sound source. The sentence has been corrected as “Therefore, we often chose a low k value to observe the general structure of acoustic events **or** a high k value to investigate the behavior of each sound source in detail.” **(Line 626)**

**RESPONSE TO REVIEWER 3**

**COMMENT:** *line 119: the definition of BSS is wrong: "A BSS model aims to learn the temporal variations of a mixture using a set of unknown sound sources." BSS is employed when there is no information about the individual sources (e.g. placement, acoustic conditions, number...) and only the joint effect (i.e. mixture) is known.* **Done.**

**REPLY:** The definition of BSS has been corrected as “A BSS model aims to separate a set of sound sources from a mixture, without prior information specific to individual sources or the mixing process.” (**Line 120**)

**COMMENT:** *line 493: "LTSA" example of one of the multiple acronyms used without definition.* **No Change.**

**REPLY:** The acronym LTSA was already explained in Line 191: “We generated a long-term spectral average (LTSA) based on 30-s and 93.75-Hz resolution.”

**COMMENT:** *The authors claim the method used here is unsupervised (e.g. line 135 or line 190) yet further down in the text we read otherwise. The "training phase to learn the source-specific spectral features" (l.544) renders the method to be at the very least semi-supervised as these spectral basis are then fixed (l.564). Further if one takes into account in that the author "checked" (l.555) whether any spectral features were mislabeled or that the "author manually annotated" (l.560) it becomes apparent that the method presented is clearly not unsupervised.* **Done.**

**REPLY:** We appreciate the constructive suggestions from the reviewers. The previous version mentioned our method as “unsupervised feature learning” in the abstract. To prevent confusion, we have removed the term “unsupervised” and rewrote this section as: “A source separation model based on the assumption of source-specific periodicity was used to factorize time-frequency representations of long-duration underwater recordings. With minimal supervision, the model learned to discriminate source-specific spectral features and prove to be effective in the separation of sounds made by cetaceans, soniferous fish, and abiotic sources from the deep-water soundscapes off northeastern Taiwan.” (**Line 27-32**)

**COMMENT:** *Equation (6) assumes the mixture to be a linear sum of sources - is this the case? Please explain why this assumption is valid in this case.* **Done.**

**REPLY:** Following the reviewer’s suggestion, we added an explanation about equation 6 in **Line 581: “**Here, we assume the mixture to be a linear sum of sources because the LTSA has been transformed to signal-to-noise ratios after the prewhitening procedure.”

**COMMENT:** *Regarding the lack of innovation: I have not found an addition or extension to the PC-NMF method presented in this text and following these comments from the authors in response to my previous review "However, parameter tuning is labor-intensive and often needs to repeat when analyzing data collected from a new environment."*

*"The application of PC-NMF can greatly reduce the labor-intensive nature of the works and the need of prior knowledge.." I am under the impression the main contribution of the presented study is the application of an existing method to new data, which I do not regard as technically innovative.* **No Change.**

**REPLY:** We appreciate the constructive suggestions from the reviewers. In ecoacoustics, the assessment of acoustic diversity and the interactions among sound sources represent a challenging task. Previous studies mostly rely on statistics-based acoustic indices to assess acoustic diversity but cannot clarify the contribution of biotic and abiotic sounds. To our knowledge, this is the first study applying the NMF-based audio source separation to assess the acoustic diversity of cetacean vocalizations, fish choruses, and anthropogenic sounds from a dataset contain multiple years of underwater recordings. Our results demonstrate the feasibility of using source separation results to investigate the potential interactions among sound sources critical for understanding the study area's biodiversity and ecosystem dynamics. As discussed in this manuscript, the applied techniques of soundscape information retrieval will advance the analysis of long-duration field recordings and facilitate the ever-growing studies of soundscape-based ecosystem monitoring.

**COMMENT:** *"The third reviewer may confuse the application of PC-NMF in different stages of data processing." -->I am indeed confused about the method in this paper yet I am very acquainted with NMF and its variants.* **No Change.**

**REPLY:** We appreciate the constructive suggestions from the reviewers. We believe that we have made the appropriate revisions according to the review comments.

**COMMENT:** *"We also added more paragraphs to demonstrate how applied techniques can improve acoustic diversity assessment and reveal the difference in spectral characteristics and phenological patterns between groups of marine soniferous animals " -->What are (the) "applied techniques"?* **Done.**

**REPLY:** The techniques applied in this study include the visualization of long-duration recordings, audio source separation, and clustering. (**Line 445**)

**COMMENT:**“*Low audio quality owing to the simultaneous recording of multiple sound sources increases clustering uncertainty.” --> I do not understand this. Simultaneous recording of multiple sound sources means that you recorded different sound sources at the same time (could mean with multiple mics). I think you mean "the recording of simultaneous sound sources". In any case, how come the sound sources being or not simultaneous affect in any way the audio quality?* **Done.**

**REPLY:** Following the review’s suggestion, we have revised this section as “However, clustering performance is subject to the selection of acoustic features and the recording of simultaneous sound sources [35]. Two signals recorded within the same time frame may distort the observed acoustic features and lead to inappropriate inductive inference.” (**Lines 115-119**)

**COMMENT:** *"**The dataset used in this study contains multiple types of biotic and abiotic sound sources that may be overlapping in time or frequency domains (Fig 2 & 3 in the revised manuscript)**, and each sound source also possesses spectral variability.* *We demonstrated that the applied technique of audio source separation not only reduces the interference among sound sources but also enables further applications, such as the variability assessment of source behaviors (Fig 4 & 7 in the revised manuscript). Most importantly, the applied technique can transform single-channel audio into multiple* *channels that* *encode the dynamics of biophony, geophony, and anthropophony. The outcomes will expand the deliverables of soundscape monitoring that are difficult to achieve by using conventional approaches."*

*--> This should be in the abstract and/or introduction as it is very clearly explained.* **Done.**

**REPLY:** Following the review’s suggestion, we added part of this section in the abstract (**Line 37**) and the final paragraph of “Marine Soundscapes Off Northeastern Taiwan” (**Line 182**) before presenting the results of this study.

**COMMENT:** *"The electrical noise in the MACHO recordings is not stationary. "*

*--> Based on the spectrograms of Figure 2, the electrical noise is certainly slow varying compared to other sources and quite obviously stationary at least four frequency bins (around 25, 30, 45 and 55kHz) from what I can see. I believe simple de-noising techniques based on Wiener filtering would remove the electrical noise if applied at a lower time scale windows (e.g. 15min instead of 11 hours).* **No Change.**

**REPLY:** We agree that some electrical noise is more predictable at a lower time scale window, such as several seconds. However, the electrical noise recorded by MACHO also contains components that are highly unpredictable (such as the noise recorded between 3 and 4.5 kHz in the two spectrograms shown below). Moreover, the spectral characteristics of these noise signals can vary among recordings (such as the noise recorded between 9 and 10 kHz in the first spectrogram). Conventional approaches based on Wiener filtering may enhance these signals and increase the difficulty of acoustic diversity assessment.
